# Psoriasis Patients Suffer From Worse Periodontal Status—A Meta-Analysis

**DOI:** 10.3389/fmed.2019.00212

**Published:** 2019-10-01

**Authors:** Pengyan Qiao, Quan Shi, Rong Zhang, Lingling E, Peihuan Wang, Juncheng Wang, Hongchen Liu

**Affiliations:** Department of Stomatology, First Medical Center, Chinese PLA General Hospital, Beijing, China

**Keywords:** psoriasis, periodontitis, periodontal health, risk factor, meta-analysis

## Abstract

**Background and Objective:** Patients with psoriasis have a significantly elevated risk of periodontitis compared with the nonpsoriasis controls. However, the data regarding the difference in the periodontal health status of the psoriasis patients and the nonpsoriasis controls are limited and inconsistent; hence, a specialized meta-analysis that quantitatively compared the periodontal status between the psoriasis and nonpsoriasis subjects by evaluating the related clinical periodontal indexes was needed. The aim of this meta-analysis was to quantitatively evaluate whether the periodontal status of psoriasis patients is worse than that of nonpsoriasis subjects.

**Methods:** We searched PubMed and EMBASE for all eligible studies that compared the periodontal status between psoriasis patients and nonpsoriasis subjects. The studies were screened based on pre-established inclusion criteria. After extracting the available periodontal indexes from the included studies, the weighted mean difference (WMD) with 95% confidence intervals (CIs) was calculated by pooling the mean and standard deviations (SD) of each index.

**Results:** In total, 8 studies, including 812 psoriasis patients and 772 nonpsoriasis subjects, were included in our meta-analysis, and the publication dates ranged from 2013 to 2019; eight periodontal indexes were analyzed. The WMD (95% CIs) for each index were: bleeding on probing (%), 9.188 (4.046–14.330, *P* < 0.001); probing depth (mm), 0.524 (0.183–0.865, *P* = 0.003); clinical attachment loss (mm), 0.408 (0.051–0.765, *P* = 0.025); plaque index, 0.186 (−0.170 to 0.543, *P* = 0.306); gingival index, 0.458 (−0.413 to 1.328, *P* = 0.303), remaining teeth, −1.709 (−2.106 to −1.312, *P* < 0.001); missing teeth, 1.130 (0.275–1.985, *P* = 0.010); the level of alveolar bone loss (mm), 0.400 (0.102–0.698, *P* = 0.008).

**Conclusion:** In summary, our meta-analysis revealed that psoriasis patients suffer from worse periodontal health than do nonpsoriasis subjects, mainly characterized by worse gingival inflammation, more alveolar bone loss, fewer remaining teeth and more missing teeth. Considering the limitations of this meta-analysis, more high-quality and well-designed studies are needed to validate our conclusions in the future.

## Introduction

As a long-lasting autoimmune disorder affecting the skin, psoriasis is characterized by scaly, red, and well-demarcated skin plaques resulting from keratinocyte hyperproliferation and altered differentiation, the presence of an inflammatory cell infiltrate, and neovascularization (1, 2) Additionally, psoriasis may develop on joints, such as elbows and knees. It is a common disease affecting 0.4–4% of the general population (3, 4). However, the exact cause of psoriasis is unclear, and it is generally believed that genetic factors, environmental risk factors and infections play important roles (5, 6). More importantly, no cure is available for psoriasis at present, and it may last lifelong, which may have a negative impact on the quality of life of the affected people. Therefore, it is necessary to better understand the complex disease mechanisms and relevant risk factors of psoriasis.

Periodontitis is a chronic infectious disease of the tooth-supporting tissues, and its main clinical manifestations are gingival bleeding, periodontal pocket formation, the destruction of connective tissue attachment, and alveolar bone loss (7, 8). As a result of complex interactions between periodontopathogens and the host's immune response, periodontitis has been shown to be associated with many immune-mediated inflammatory diseases, such as chronic obstructive pulmonary disease (COPD) (9), chronic kidney diseases (10), and rheumatoid arthritis (11). Interestingly, possible links between psoriasis and periodontitis have also been studied (12, 13). Not only do both psoriasis and periodontal disease involve an exaggerated immune response in epithelial surfaces and a dysregulation of the host inflammatory response but they also share common risk factors.

Several epidemiological studies have noted that patients with psoriasis have a significantly elevated risk of periodontitis compared with nonpsoriasis controls; this is especially true in patients with severe psoriasis and psoriatic arthritis (14–19). In addition, a meta-analysis reported that patients with periodontitis have a significantly increased risk for psoriasis (12). However, the number of studies included in that meta-analysis was limited. Based on the current evidence, there are also some conflicting results (20). Moreover, although the association has been shown in a number of observational studies, the data regarding the difference in periodontal health status between the psoriasis patients and the nonpsoriasis controls are limited, and individual studies are often small and may provide imprecise estimates. Therefore, a specialized meta-analysis that quantitatively compares the periodontal status between the psoriasis and nonpsoriasis subjects by evaluating the related clinical periodontal indexes is needed.

We hypothesized that psoriasis patients suffer from worse periodontal health. Therefore, we performed the current meta-analysis to identify all related clinical studies comparing periodontal status indexes between psoriasis patients and nonpsoriasis subjects and then to evaluate whether the periodontal status of psoriasis patients is worse than that of the nonpsoriasis subjects by comparing the clinical periodontal parameters. Through this research, we hope to facilitate a better understanding of the association between psoriasis and periodontal health as well as to provide better evidence-based evaluations and recommendations to clinicians and patients.

## Methods

### Search Strategy and Selection Criteria

For this meta-analysis, we searched PubMed and EMBASE with no language or publication date restrictions on May 20, 2019, to obtain all of the relevant clinical studies for inclusion in our study. The following combination of key words was used: (“periodontitis” OR “periodontal disease”) AND (psoriasis). We additionally searched the reference lists of the included studies, relevant reviews and meta-analyses for additional potential studies.

Following the searches of the electronic databases, studies were selected based on the pre-established inclusion criteria. A study could be included in this meta-analysis if all of the following conditions were met: (1) the study compared the periodontal status between the psoriasis patients and nonpsoriasis subjects; (2) the psoriasis patients were described clearly; (3) data regarding the periodontal indexes were available and could be extracted; and (4) the studies used validated methods to perform the periodontal examinations. However, the articles were excluded if (1) they were reviews, comments, letters or case reports; (2) they were *in vitro* or animal studies; (3) the study lacked the necessary data or a nonpsoriasis control group; or (4) the periodontal indexes could be affected by other interventions.

Study screening was performed by two independent investigators (QP and SQ) based on the criteria above, and a third investigator (WJ) arbitrated when any conflict occurred regarding the suitability of a study for inclusion. At first, the investigators screened all titles and abstracts independently to exclude the duplicates and obviously irrelevant articles. Second, we examined the full texts of all the remaining papers and assessed them for their adherence to prespecified inclusion criteria. Finally, only the articles meeting the inclusion criteria were included in this meta-analysis.

### Study Quality Assessment and Data Extraction

The following information was independently extracted from the included studies by two investigators (ZR and EL): the name of the first author; year of publication; study location (country); numbers, ages, and sexes of participants in psoriasis group and nonpsoriasis group; periodontal parameters; and the results, including the level of alveolar bone loss (ABL), bleeding index (BI), bleeding on probing (BOP), clinical attachment loss (CAL), gingival index (GI), probing depth (PD), plaque index (PI), and the number of remaining or missing teeth.

Study quality was assessed with the Newcastle-Ottawa Quality Assessment Scale in the current meta-analysis (21); the Newcastle-Ottawa Scale mainly evaluates the included studies on the basis of the method of subject selection (0–4 points), comparability of the study subjects (0–2 points), and outcome of exposure (0–3 points). Therefore, the NOS score ranges from 0 to 9, while scores of 0 to 3, 4 to 6, and 7 to 9 indicate low, moderate, and high study quality, respectively. All included studies were assessed by two investigators (QP and SQ) independently, and discrepancies were resolved by discussion with a third investigator (WJ).

### Statistical Analysis

The data analyses were performed with STATA software (Version 12.0; Stata Corp, College Station, Texas, USA). Because each available periodontal index in the current study was continuous, the weighted mean difference (WMD) with 95% confidence intervals (CIs) was calculated by pooling the means and standard deviations (SD) of each index from all studies, by which we aimed to explore the difference in the periodontal status between the psoriasis group and the nonpsoriasis group. In addition, we assessed the heterogeneity across studies using the *I*^2^ statistic: if the *I*^2^ value was <25%, it suggested that the heterogeneity was low, and a fixed effect model was adopted to estimate the WMD and 95% CI; otherwise, a random effects model was used. Publication bias was assessed by visualization of a plot of Egger's publication bias plot and *P*-value of Egger's test if the number of the studies were more than five in each periodontal indexes. A *P*-value < 0.05 was considered statistically significant.

## Results

### Summary of the Included Studies

**Figure 1:**
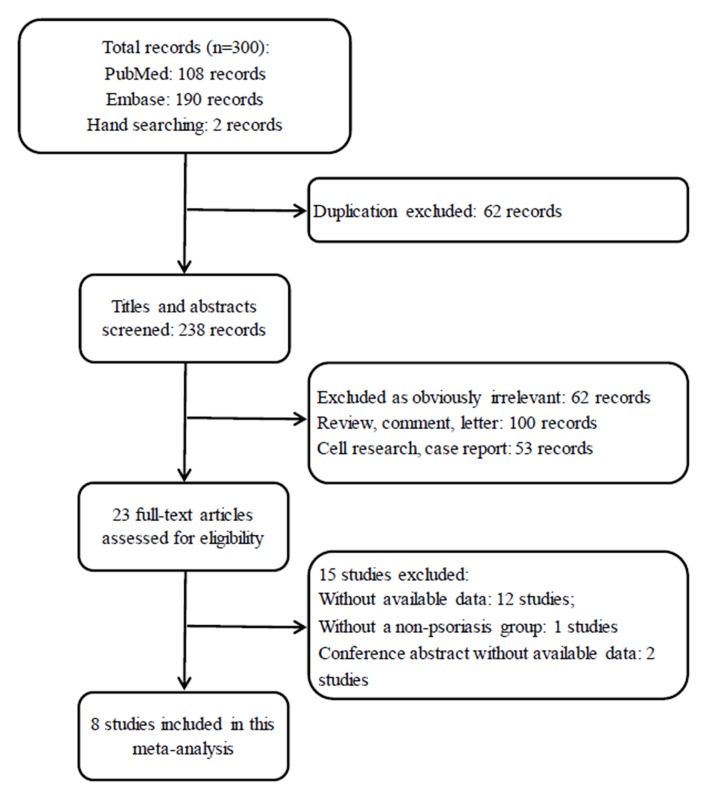
Flow chart for the study selection process.

In total, 300 potentially relevant articles were identified after our initial search of PubMed, EMBASE and the references of relevant articles. After excluding the duplicated records, 238 studies were left for screening. Next, 215 records that were irrelevant to our topic were excluded after the titles and abstracts were screened, leaving 23 articles for a further full text review. Finally, 8 studies fulfilled the eligibility criteria and were included in this meta-analysis (20, 22–28). The flow chart of the study selection process is displayed in Figure 1.

The publication dates of the 8 included studies ranged from 2013 to 2019. In total, 812 psoriasis patients and 772 nonpsoriasis subjects were studied. Two studies (25, 28) were conducted in Asia, five were conducted in Europe (20, 23, 24, 26, 27) and one was conducted in South America (22). In total, data pertaining to eight indexes were extracted and analyzed in our meta-analysis: BOP, PD, CAL, PI, GI, ABL, remaining teeth, and missing teeth. The publication year, country, information about the psoriasis patients and controls, and the available periodontal indexes from each included study are shown in Table 1.

**Table 1:** Characteristics of the included studies.

**References**	**Nation**	**Psoriasis group**		**Non-psoriasis group**		**Available periodontal indexes**	**NOS score**
		**Number (male/female)**	**Age (mean ± SD)**	**Number (male/female)**	**Age (mean ± SD)**		
Mendes et al. (22)	Brazil	397 (159/238)	46.03 ± 8.34	359 (119/240)	47.47 ± 5.06	Remaining teeth; BOP; PD; CAL	8
Woeste et al. (23)	Germany	100 (59/41)	47.4 ± 14.7	101 (43/58)	46.9 ± 16.8	BOP; missing teeth	6
Sezer et al. (24)	Turkey	80 (43/37)	42.88 ± 11.50	40 (20/20)	42.28 ± 11.86	PD; CAL; PI; BOP	6
Sharma et al. (25)	India	33 (19/14)	34.58 ± 3.47	35 (18/17)	34.34 ± 3.11	PD; CAL	6
Skudutyte-Rysstad et al. (26)	Norway	50 (38/12)	44.4 ± 10.2	121 (61/60)	48.6 ± 9.4	Missing teeth; BOP	7
Üstün et al. (20)	Turkey	51 (24/27)	41.73 ± 11.27	50 (24/26)	37.90 ± 11.16	CAL; PD; PI; GI	6
Fadel et al. (27)	Sweden	89 (46/43)	59 ± 10	54 (21/33)	60 ± 11	Remaining teeth; BOP; ABL	6
Mayer et al. (28)	Israel	12 (5/7)	48.7 ± 10.4	12 (5/7)	51.5 ± 9.97	PD; GI; PI;BOP	5

*SD, standard deviation; NOS, Newcastle-Ottawa Scale; ABL, level of alveolar bone loss; BOP, bleeding on probing; CAL, clinical attachment loss; GI, gingival index; PD, probing depth; PI, plaque index*.

For the study quality assessment, two studies had ≥7 points (22, 26) and were regarded as high quality, and six studies had scores ranging from 4 to 6 points (20, 23–25, 27, 28), indicating that they were of moderate quality. No studies included in the meta-analysis were of low quality. The mean NOS score was 6.25. The quality assessments of the included studies are also shown in Table 1.

### Data Analysis

#### Bleeding on Probing

**Figure 2:**
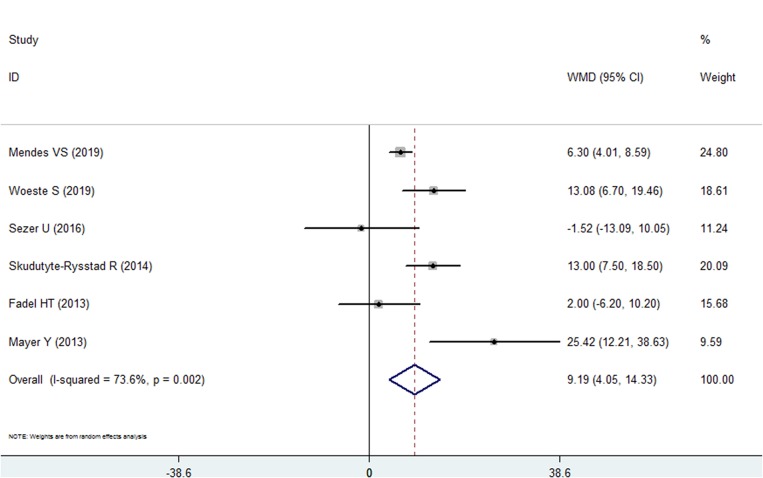
Forest plot of the weighted mean difference in bleeding on probing between the psoriasis and nonpsoriasis groups.

Six of the included studies reported BOP data in the psoriasis and nonpsoriasis group (22–24, 26–28). The results of our pooled analysis revealed that BOP (%) was found in a higher proportion of the psoriasis patients than in the nonpsoriasis subjects, and the weighted mean difference was 9.88, which is statistically significant (95% CI: 4.046–14.330, *P* < 0.001). However, high heterogeneity (*I*^2^ = 93.8%) between the studies was found; therefore, a random effects model was selected (Figure 2, Table 2). No significant publication bias was identified in this comparisons by Egger's test with a *P*-value of 0.493 (Supplementary Figure 1).

**Table 2:** Summary of the periodontal indexes analyzed in this meta-analysis.

**Index**	**Number**	**WMD**	**95% CI**	***P***	***I*^**2**^ (%)**
BOP	6	9.188	4.046 to 14.330	<0.001	73.6
PD	5	0.524	0.183 to 0.865	0.003	86.8
CAL	4	0.408	0.051 to 0.765	0.025	81.0
PI	3	0.186	−0.170 to 0.543	0.306	72.1
GI	2	0.458	−0.413 to 1.328	0.303	92.3
Remaining teeth	2	−1.709	−2.106 to −1.312	<0.001	0
Missing teeth	2	1.130	0.275 to 1.985	0.010	0
ABL	1	0.400	0.102 to 0.698	0.008	–

*WMD, weighted mean difference; CI, confidence intervals; BOP, bleeding on probing; PD, probing depth; CAL, clinical attachment loss; PI, plaque index; GI, gingival index; ABL, level of alveolar bone loss*.

#### Probing Depth

**Figure 3:**
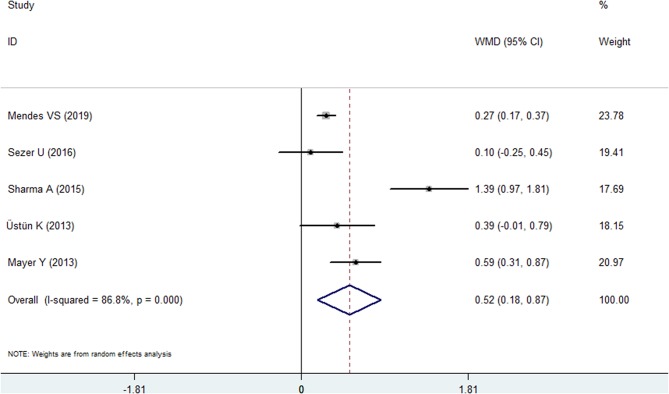
Forest plot of the weighted mean difference in probing depth between the psoriasis and nonpsoriasis groups.

Five included studies reported PD data for the psoriasis and nonpsoriasis groups (20, 22, 24, 25, 28). The results of meta-analysis revealed that the PD was deeper in the psoriasis patients than in the control group, and the difference in the means was 0.524 mm (95% CI: 0.183–0.865, *P* = 0.003, *I*^2^ = 86.8%, random effects model, Figure 3, Table 2). Similar to BOP, the Egger's test analysis failed to found a publication bias in PD, either (*P* = 0.311, Supplementary Figure 2).

#### Clinical Attachment Loss

**Figure 4:**
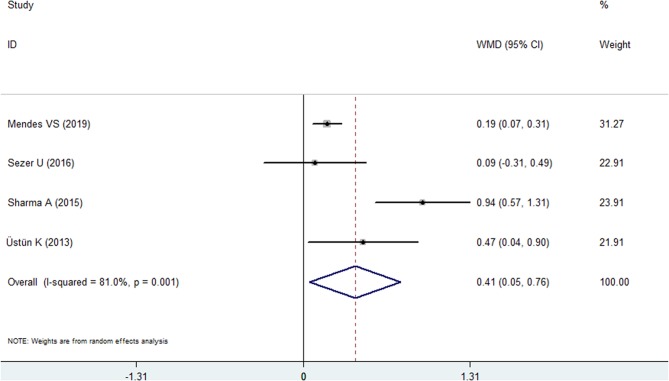
Forest plot of the weighted mean difference in clinical attachment loss between the psoriasis and nonpsoriasis groups.

Four included studies reported the CAL index for the psoriasis and nonpsoriasis groups (20, 22, 24, 25). The overall effects found more clinical attachment loss in the psoriasis patients than in the control group. The WMD was 0.408 mm (95% CI: 0.051–0.765, Figure 4, Table 2), which was statistically significant (*P* = 0.025). A random effects model was used because of high heterogeneity (*I*^2^ = 81.0%).

#### Plaque Index

**Figure 5:**
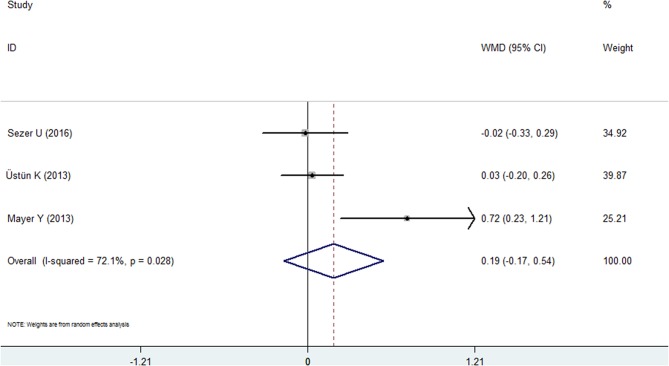
Forest plot of the weighted mean difference in plaque index between the psoriasis and nonpsoriasis groups.

Three studies reported the results of the PI in both groups (20, 24, 28). However, no statistical significance was found in this comprehensive analysis. The WMD was 0.186 (95% CI: −0.170 to 0.543, Figure 5, Table 2), and the *P*-value was 0.306. A random effects model was used because of high heterogeneity (*I*^2^ = 72.1%).

#### Gingival Index

**Figure 6:**
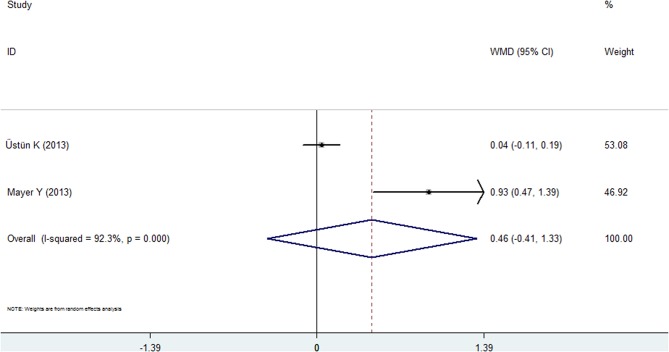
Forest plot of the weighted mean difference in gingival index between the psoriasis and nonpsoriasis groups.

Two studies reported the results of the GI for the psoriasis and nonpsoriasis groups (20, 28). The pooled analysis failed to reveal statistical significance: the WMD was 0.458 (95% CI: −0.413 to 1.328, random effects, *I*^2^ = 92.3%, Figure 6, Table 2), and the *P*-value was 0.306.

#### Remaining Teeth and Missing Teeth

**Figure 7:**
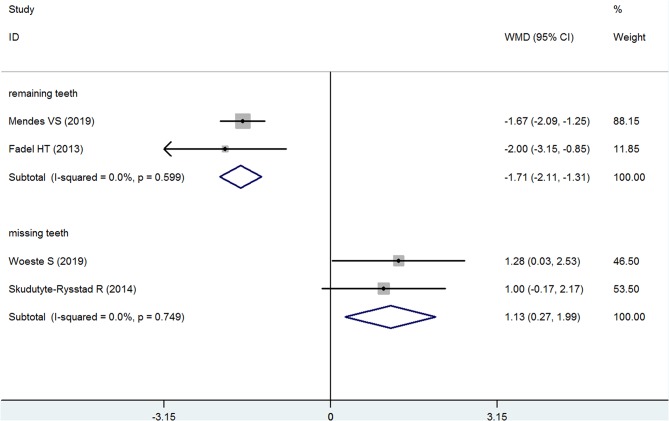
Forest plot of the weighted mean difference in remaining teeth and missing teeth between the psoriasis and nonpsoriasis groups.

Two studies each reported the results for remaining teeth (22, 27) and missing teeth (23, 26) in the psoriasis and nonpsoriasis groups. The pooled analyses of these two indexes are consistent, and the results indicate that the psoriasis group had fewer remaining teeth and had more missing teeth than the nonpsoriasis subjects. For the remaining teeth, the mean difference was −1.709, which was significant (95% CI: −2.106 to −1.312, *P* < 0.001, fixed effects model, *I*^2^ = 0%, Figure 7), and the WMD for missing teeth was 1.130 (95% CI: 0.257–1.985, *P* = 0.010, fixed effects model, *I*^2^ = 0%, Figure 7, Table 2).

#### Level of Alveolar Bone Loss

Only one study (27) reported the assessment of the level of alveolar bone loss in the psoriasis and nonpsoriasis groups; therefore, we did not generate a forest diagram. More alveolar bone loss was found in the psoriasis group. The mean difference was 0.400 (95% CI: 0.102–0.698, *P* = 0.008, Table 2).

## Discussion

There has been increasing interest in the association between periodontal disease and chronic inflammatory diseases or systemic diseases, although the underlying nature of the association is not fully understood (29, 30). Some case-control studies or cohort studies concluded that periodontitis has an association with psoriasis (16, 19); however, the number of those studies is limited, and the periodontal health status difference between the psoriasis and nonpsoriasis groups has not been systematically studied. Because many periodontal indexes are used in the clinic to evaluate the inflammation levels and changes in soft and hard tissue, such as the BOP, GI, and CAL, and a single study may not be comprehensive, a specialized meta-analysis that quantitatively compares the periodontal status between the psoriasis and nonpsoriasis subjects was needed.

In total, we included 8 studies in this meta-analysis (20, 22–28), with 812 psoriasis patients and 772 nonpsoriasis controls. All of these studies were of moderate-high quality. In the included studies, 8 available periodontal indexes were studied: BOP, PD, CAL, PI, GI, remaining teeth, missing teeth and ABL. Our pooled analyses revealed that among these 8 available periodontal indexes, 6 indexes (BOP, PD, CAL, remaining teeth, missing teeth and ABL) confirmed that psoriasis patients suffer from worse periodontal health, including worse gingival inflammation, more alveolar bone loss, fewer remaining teeth and more missing teeth. For the PI and GI, our analysis failed to show a statistically significant difference, but the number of the articles that reported those two indexes were limited (3 and 2 studies, respectively). Moreover, the Egger's test with plots and *P*-values of BOP and PD both failed to found a publication bias.

Several prospective studies have explored the relationship between periodontitis and psoriasis, some of which also had similar results to those in our current study. Egeberg et al. investigated the association between psoriasis and periodontitis in a nationwide cohort study, and they found a significant increase in the risk of periodontitis that was associated with psoriasis, especially in patients with severe psoriasis and psoriatic arthritis (15) An increased multivariate risk of psoriasis was observed for those with mild periodontal bone loss and moderate-to-severe periodontal bone loss compared to those without periodontal bone loss (16). In addition to prospective studies, cross-sectional studies and case-control studies are often applied to investigate periodontal status. Sarac et al. found that psoriasis patients' periodontal health was worse than that of the healthy controls, mainly in terms of a significantly higher community periodontal index (CPI) score and worse oral hygiene (31). After comparing psoriasis patients with age- and sex-matched controls, the psoriasis patients were shown to have a significantly lower radiographic bone level and a significantly greater number of missing teeth (19). Moreover, some related studies also concluded that periodontal status was associated with the severity of psoriasis (15, 25).

Our meta-analysis results supported our hypothesis, namely, psoriasis patients suffer from worse periodontal health. Based on previous reports, despite the uncertainty regarding the specific mechanism by which periodontitis and psoriasis interact, several factors may contribute to the worse periodontal status of psoriasis patients. First, infection may play a role. Periodontitis is a chronic infectious disease of the tooth-supporting tissues. Some types of microbes play important roles in the occurrence and development of the disease (32, 33), such as *Actinobacillus actinomycetemcomitans, Porphyromanas gingivalis*, and *Tannerella forsythia*. These pathogenic bacteria that can cause periodontitis also have some effects on other diseases. For example, *P. gingivalis* can colonize the brain and play a central role in the pathogenesis of Alzheimer's disease (34). Infections are also well-known triggers for psoriasis (6, 35). More importantly, there have been studies that have shown an increased concentration of bacterial DNA in the synovial fluid of patients with psoriatic arthritis compared to controls (36). However, the role of oral bacteria in the two diseases needs further research. Second, immune dysregulation and common pathophysiological processes may be involved. It has been found that periodontitis and psoriasis share some common immunopathogenic processes (37–40), such as the activation and deregulation of the innate and adaptive immune responses. Therefore, the immune response and related effects (such as activated immune cells and inflammatory mediators) caused by psoriasis can make an individual susceptible to periodontal tissue destruction. In contrast, oral pathogens and inflammatory cytokines from periodontal lesions induce systemic inflammation, which may also contribute to the pathogenesis of psoriasis. Third, common risk factors and comorbidities may play roles in the association. Psoriasis and periodontitis share common risk factors (14, 41), for example, smoking, stress, and obesity. A strong association has been found between smoking and both diseases. In addition, many diseases, including diabetes (42, 43) and COPD (9, 44), cardiovascular diseases (CVD) (45, 46), obesity (47), may interact with both diseases at the same time, but the related mechanisms are still not completely clear, and comorbidities often tend to be more frequently seen in severe disease (47). Therefore, in psoriasis patients, the existence of risk factors and comorbidities could not only affect the occurrence and development of psoriasis but also accelerate the destruction of periodontal tissue.

In order to give a clear and reliable answer to the periodontal status difference between the psoriasis and nonpsoriasis patients, it is crucial to control confounding factors. As we have mentioned above, many factors, such as smoking, age, gender, drinking and some disease such as diabetes, obesity, COPD, are the common risk factors between periodontitis and psoriasis. It is undeniable that these confounding factors may affect the occurrence, development and treatment of these two disease. For example, smoking could not only affect the periodontal status, but also affect oxidative stress, interaction with active signaling pathways in psoriasis and vascular influences (48–50). As for determine the causal relationship, controlling and analyze these confounding factors is of great importance. Currently, this issue has attracted the attention of researchers and several studies have conducted preliminary research on these factors (15, 22, 23). Despite our main concern is the correlation between the psoriasis and worse periodontal health, an in-depth analysis by control these factors could help to give a better and in-depth understanding. However, in our current meta-analysis, the included studies in our meta-analysis, they have provided different and insufficient data. Because of the limited information obtained from the included studies, we failed to conduct a subgroup analysis or the a meta-regression analysis to explore these risk factors in depth. Therefore, we call for more related studies in the future.

Unfortunately, despite our meta-analysis and related clinical studies revealing that psoriasis patients suffer from worse periodontal health, whether active treatment of periodontal disease contributes to improved outcomes for psoriasis patients has not yet been studied. Keller et al. found that treatment for periodontitis could attenuate the risk for subsequent psoriasis but could not eliminate the risk completely (17). Considering the lack of an effective cure and high annual treatment costs for psoriasis, it is important to conduct relevant research in the future.

To the best of our knowledge, this is the first meta-analysis to specifically compare the periodontal statuses of psoriasis patients and nonpsoriasis subjects, by which we aimed to provide more evidence regarding the association between psoriasis and periodontal disease. However, we must acknowledge that there are limitations in our meta-analysis. First, the number of studies that reported each periodontal index was limited. For four indexes (GI, remaining teeth, missing teeth, ABL), there were fewer than 3 studies. Second, the comprehensive analysis revealed high levels of heterogeneity for some indexes; therefore, random effects models were adopted. Third, because of the limited study number included in our meta-analysis, we did not perform subgroup analyses or meta-regression analysis to explore related risk factors. Therefore, the findings should be interpreted with caution, and well-designed studies are needed in the future.

In summary, our meta-analysis supports the hypothesis that psoriasis patients suffer from worse periodontal health compared with nonpsoriasis subjects, mainly including worse gingival inflammation, more alveolar bone loss, fewer remaining teeth and more missing teeth. These results indicate that we must pay more attention to periodontal health during the prevention, occurrence, and treatment of psoriasis. In addition, we call for further research about the effect of the treatment of periodontal disease on psoriasis. Considering the limitations of this meta-analysis, more high-quality and well-designed studies are needed to validate our conclusion in the future.

## Data Availability Statement

All datasets generated for this study are included in the manuscript/Supplementary Files.

## Author Contributions

PQ and QS: literature search, study selection, and risk of bias evaluation. RZ, LE, and PW: data extraction and data analysis. PQ, QS, RZ, and JW: manuscript drafting and revision. HL and JW are the corresponding authors, and they undertook the work of designing this meta-analysis. All authors read and approved the final manuscript.

### Conflict of Interest

The authors declare that the research was conducted in the absence of any commercial or financial relationships that could be construed as a potential conflict of interest.

## Supplementary Material

Supplementary Figure 1 Egger's publication bias plot of bleeding on probing.

Supplementary Figure 2 Egger's publication bias plot of probing depth.
