# Supplementary figures and images for: Psoriasis Patients Suffer From Worse Periodontal Status—A Meta-Analysis
Source: Front Med (Lausanne). 2019 Oct 1;6:212. doi: 10.3389/fmed.2019.00212 (PMC6779717; doi:10.3389/fmed.2019.00212)

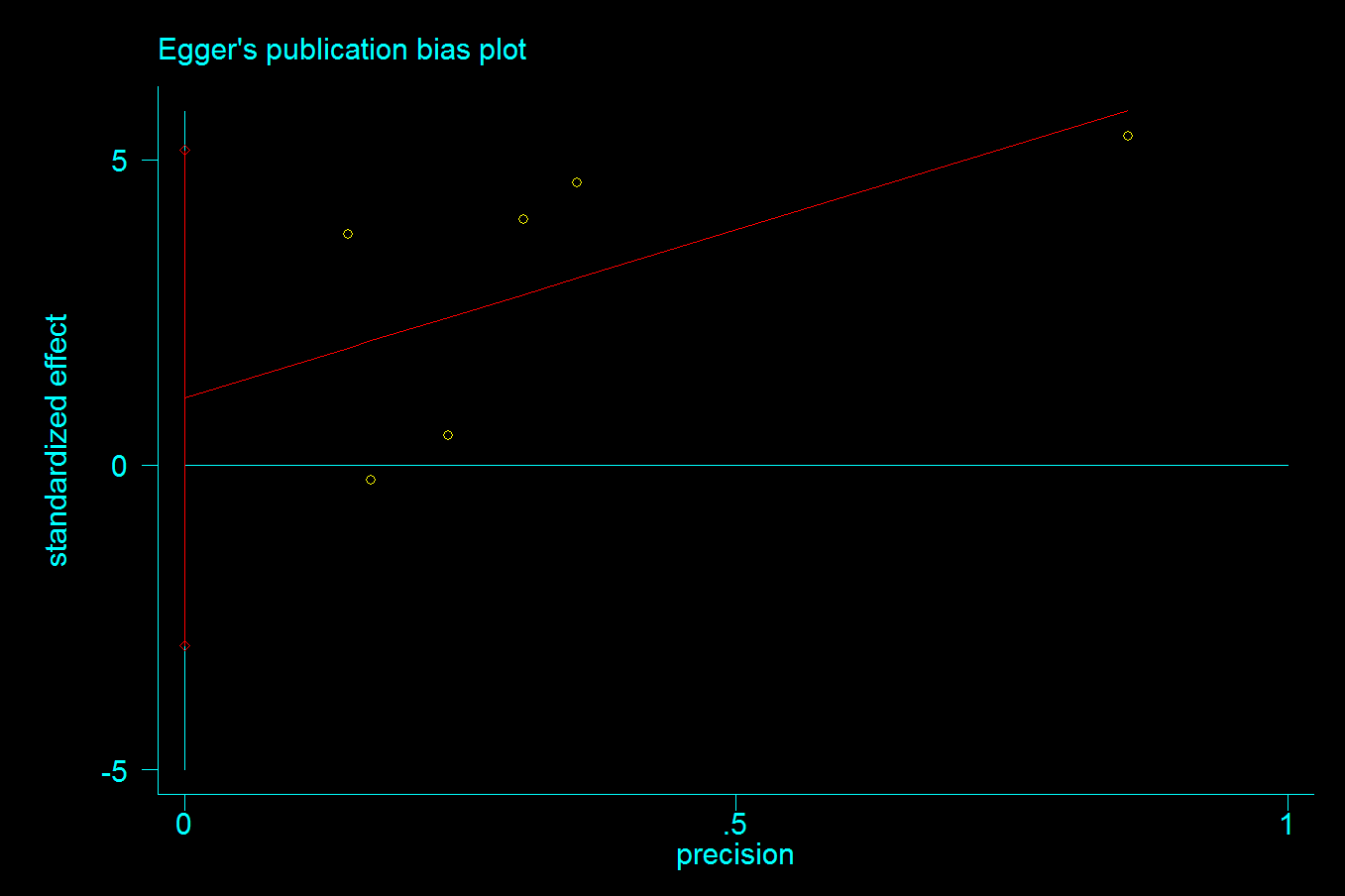

Supplement: Supplementary Figure 1 — Egger's publication bias plot of bleeding on probing. [file Image_1.TIF]

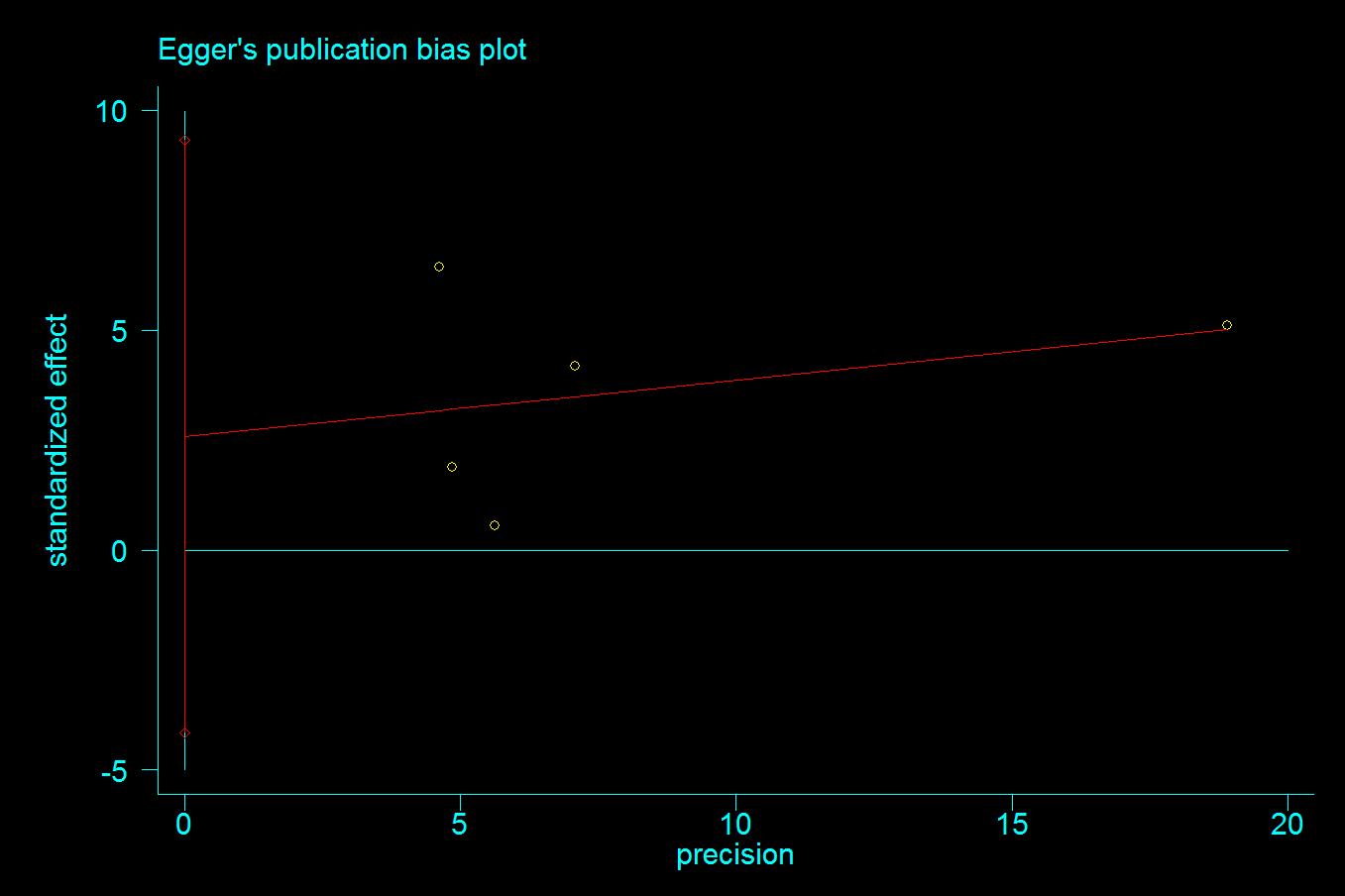

Supplement: Supplementary Figure 2 — Egger's publication bias plot of probing depth. [file Image_2.TIF]
